# Predictions of the Chinese Forest Frog (*Rana chensinensis*) Distribution Pattern Under Climate Change up to 2090s

**DOI:** 10.3390/biology14070754

**Published:** 2025-06-24

**Authors:** Ying Fu, Juanjuan Lu, Pinhong Yang, Jie Pi

**Affiliations:** Key Laboratory of Featured Aquaculture, Hunan Applied Technology University, Changde 415100, China; 18570148357@163.com (Y.F.);

**Keywords:** *Rana chensinensis*, climate change scenarios, MaxEnt and Biomod2 models, environmental variable, potential geographic distribution

## Abstract

The Chinese forest frog (*Rana chensinensis*) has high ecological and economic value and is an ecologically important species that is very sensitive to environmental changes. However, climate change and increasing human activity are posing growing threats to its natural habitat. To address these challenges, this study aimed to predict the current and future geographic distribution of the Chinese forest frog and to identify the environmental drivers influencing its habitat suitability. Here, the optimized MaxEnt and Biomod2 were used to assess 127 species occurrence records and 22 environmental variables and model and analyze changes in the geographic distribution of the Chinese forest frog in different periods. The environmental factors underlying geographical distribution changes and migration trends in distribution areas under climate change were assessed. The optimized MaxEnt model generated the best predictions, showing that the current most highly suitable areas are located in Chongqing, Sichuan, and Gansu provinces. The optimized MaxEnt model demonstrated exceptionally high predictive accuracy, with mean AUC values of 0.968 ± 0.000 and TSS values of 0.800 ± 0.089. The total area of the current potentially suitable habitat was estimated at 426 × 10^4^ km^2^. The principal ecological factors influencing the distribution of the Chinese forest frog are Bio9, Bio10, and human activity. Under future scenarios, the potentially suitable habitat for the Chinese forest frog is projected to shift westward and southward. Compared to the current situation, the potentially suitable habitat for the Chinese forest frog is expected to significantly shrink across all four future scenarios (2050s-SSP126, 2050s-SSP585, 2090s-SSP126, and 2090s-SSP585), posing serious threats to its survival. This study not only identifies the key ecological factors limiting the potential distribution of the Chinese forest frog but also provides a scientific basis and data support for the development of conservation strategies and habitat restoration efforts.

## Simple Summary

The Chinese forest frog is an amphibian of significant ecological and economic value, exhibiting a high sensitivity to environmental changes. In this study, an optimized MaxEnt model is employed to assess the current and future habitat suitability of this species under different climate scenarios. The results indicate that suitable habitats for the Chinese forest frog will gradually shrink and shift southward and westward as climate change progresses. These insights provide a reference for conservation strategies for this ecologically vulnerable species.

## 1. Introduction

Climate change significantly impacts species, influencing both population dynamics and migration patterns [1]. As global temperatures rise, species must adapt or migrate poleward/upward to track suitable thermal niches [2,3]. Predicting species’ responses to these changes is a central objective for conservation biologists [4]. Understanding the relationship between the climate and species’ geographic distributions allows for identifying limiting factors, predicting potential shifts, and understanding underlying causes.

Amphibians, which are highly sensitive to environmental changes, serve as critical indicators of climate change and are often considered “early warning systems” [5]. The effects of climate change on amphibians are diverse and include changes in body size [6,7,8], range, and population decline [9,10]. Over the past five decades, amphibians have faced numerous threats, with population declines occurring faster than those of birds and mammals, and extinction rates surpassing those of other vertebrates [11,12]. Whilst the decline in amphibian populations is widely acknowledged, various hypotheses have been proposed to explain this phenomenon, including the effects of invasive species, habitat fragmentation, emerging diseases, and synergistic climate effects [5,13]. However, no single explanation has gained universal acceptance [14].

Species distribution models (SDMs) have become vital tools in ecology and biogeography for assessing the impacts of climate change on future species distributions and biodiversity patterns [15]. Various SDMs have been extensively applied in ecological research, including the generalized linear model (GLM) [16], the genetic algorithm for rule-set production (GARP) [17], the maximum entropy model (MaxEnt) [18], the random forest model (RF) [19], and the BIOCLIM model [20]. The MaxEnt model has been widely used in species conservation and biological control due to its capability to model species distributions with limited occurrence records and to incorporate both quantitative (e.g., temperature and precipitation) and qualitative (e.g., land cover type and soil category) environmental variables [21]. In recent years, an increasing number of species distribution studies have employed the Biomod2 ensemble modeling approach for a variety of applications, including species distribution predictions, evaluations of the impacts of climate change on future distributions, assessments of habitat suitability, and contributions to the management strategies for invasive species [22]. This approach provides crucial insights for scientists and policymakers, facilitating the development of strategies to mitigate the adverse effects of climate change on global biodiversity.

*Rana chensinensis* (Chinese forest frog), a frog species endemic to and widely distributed in China, belongs to the genus *Rana* within the family *Ranidae* [23,24]. It is classified as a second-class protected species under the Chinese “three protected animals” list and is designated as “Least Concern” on the latest IUCN Red List [25]. Due to its high economic and medicinal value, the population of the Chinese forest frog has declined rapidly, raising concerns about its survival in the wild [26].

To scientifically elucidate the responses of the potential geographic distribution of the Chinese forest frog to climate change across different time periods, the parameter settings of the MaxEnt model (version 3.4.1) were optimized using the ENMeval (v.2.0.5) package to enhance predictive performance. Concurrently, the Biomod2 platform (version 3.3-7) was used to construct an ensemble model integrating four commonly applied algorithms—generalized linear models (GLMs), generalized boosted regression models (GBMs), generalized additive models (GAMs), and random forest (RF)—to improve prediction accuracy and model robustness. The performance of the MaxEnt and Biomod2 ensemble models was then evaluated and compared under various climate change scenarios. Finally, the optimized models were applied to simulate and analyze the potential suitable habitats of the Chinese forest frog. The objectives of this study were (1) to identify the key environmental factors restricting the species’ potential distribution and (2) to provide a scientific foundation for conservation strategies and habitat restoration efforts targeting the Chinese forest frog.

## 2. Materials and Methods

### 2.1. Species Occurrence Data Collection and Processing

**Figure 1:**
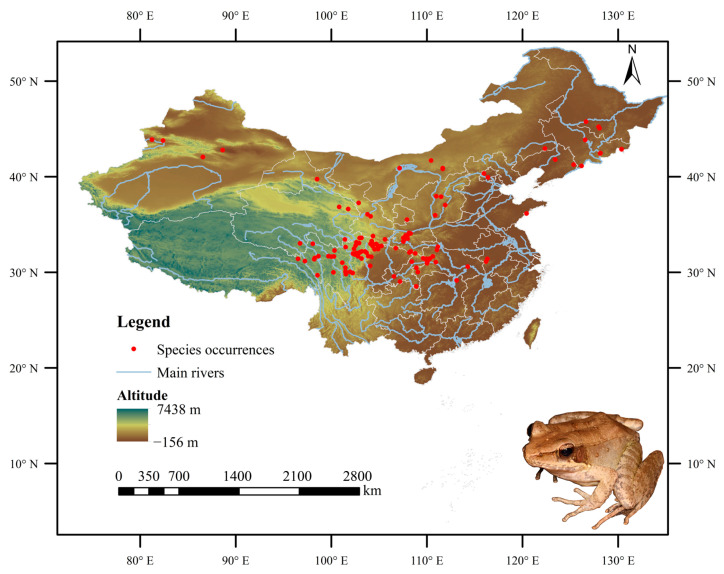
Confirmed coordinate distribution of the Chinese forest frog in China.

In this study, a total of 2905 occurrence records for the Chinese forest frog were obtained from the Global Biodiversity Information Facility (GBIF; available online: https://www.gbif.org (accessed on 29 August 2024)), one of the most widely used open-access biodiversity databases in ecological research. The “spThin” package was used to remove the samples that were clustered within 1 km to reduce the sampling deviation impact [27]. For the accuracy of model prediction, only one distribution point was kept for each grid (1 km × 1 km). Ultimately, after filtering to retain only coordinate points within China and eliminating records with high spatial autocorrelation, missing data, and duplicates, 127 valid occurrence records were preserved for modeling. The corresponding distributional locations are illustrated in Figure 1.

### 2.2. Sources and Processing of Environmental Data

A total of 22 environmental variables were selected based on the ecological characteristics of the Chinese forest frog, including 19 bioclimatic variables, elevation, human footprint, distance to roads and waterways, and the normalized difference vegetation index (NDVI) (Table 1). All environmental data layers were resampled to a spatial resolution of 2.5 arc-minutes, projected to the GCS-WGS-1984 coordinate system, and converted into an ASCII (*.asc) format using ArcGIS 10.8. Elevation and bioclimatic variables for the current period (1970–2000), the 2050s (2041–2060), and the 2090s (2081–2100) were obtained from WorldClim (available online: http://www.worldclim.org/ (accessed on 10 September 2024)). Future climate scenarios were derived from the BCC-CSM2-MR model (Beijing Climate Center Climate System Model version 2-Medium Resolution) under SSP126 and SSP585, representing the lowest and highest greenhouse gas emission trajectories in IPCC AR6. As a CMIP6-endorsed global climate model, BCC-CSM2-MR was selected for its demonstrated accuracy in simulating East Asian monsoon systems, a critical factor for regional precipitation and temperature projections [28]. Human footprint data were sourced from the Socioeconomic Data and Applications Center (SEDAC; available online: https://sedac.ciesin.columbia.edu (accessed on 10 September 2024)). Distances to roads and waterways were calculated using Euclidean analysis in ArcGIS based on spatial data from OpenStreetMap (available online: https://www.openstreetmap.org (accessed on 10 September 2024)). The NDVI was calculated as the average annual value from 2010 to 2019, using datasets from the Resource and Environment Data Center of the Chinese Academy of Sciences (available online: https://www.resdc.cn/ (accessed on 10 September 2024)). Administrative boundary data were also retrieved from the same source.

To minimize the impact of multicollinearity among environmental variables, Pearson’s correlation coefficients (|r|) were calculated using the ‘ENMTools’ package in R (v4.1.3) [3,29,30]. When the correlation between any two variables exceeded 0.7, the variable contributing more to the ecological response was retained. Based on this criterion, 10 variables were ultimately selected from the original set of 24 for model construction: Bio3 (isothermality), Bio4 (temperature seasonality), Bio9 (mean temperature of the driest quarter), Bio10 (mean temperature of the warmest quarter), Bio12 (annual precipitation), Bio15 (precipitation seasonality), elevation, human footprint, distance to roads and waterways, and NDVI.

### 2.3. Species Distribution Modeling and Model Optimization

MaxEnt (v 3.4.1) software was used for model construction. The organized species point information of the Chinese forest frog was saved in a CSV data format and imported into the software together with the environmental variables. The data were randomly divided: 75% of the location point data were used for training the model, and the remaining 25% were used for validating the MaxEnt model. All other parameters were set to their default values [31].

To address potential sampling bias and spatial heterogeneity in the occurrence records, particularly those derived from online databases, a bias file was generated for MaxEnt using the Gaussian kernel density estimation approach implemented via the ENMeval (v.2.0.5) in R (v4.1.3) [32]. This bias layer assigned higher weights to presence-only points located in sparsely sampled regions, while background points were preferentially drawn from areas with denser sampling intensity. This correction strategy helps reduce model overfitting and improves prediction accuracy [32,33]. To minimize potential bias as much as possible, the optimal feature combination was selected using the ENMeval (v2.0.5) package, resulting in the final choice of LQHTP (delta AICc = 0) [34,35]. The regularization multiplier was set to 1. The final MaxEnt model was built using MaxEnt software version 3.4.1 with the selected parameters. The other settings remained consistent. To reduce the uncertainty of the MaxEnt model, the modeling process was repeated 10 times [3].

The second step involved creating the model using the Biomod2 (v3.3-7) package. Four algorithms were applied in this study: GLMs, GBMs, GAMs, and RF [30]. The algorithms used the Biomod2 default model settings [36]. As with the MaxEnt model, the occurrence data were randomly divided into two groups. In addition, to improve the simulation of the actual distribution and reduce the spatial variation, 1000 pseudo-absence points were systematically selected, and this procedure was repeated three times to ensure the robustness of the model.

### 2.4. Evaluation of Modeling

In this study, the values of TSS and the area under the curve (AUC) were selected to evaluate the predictive ability of the model. TSS is the true skill statistic, whose value ranges from −1 to 1. The closer the value is to 1, the better the predictive ability of the model [3]. The AUC takes values ranging from 0 to 1, with larger values indicating better prediction results: 0.9 to 1.0 indicates very good results, 0.8 to 0.9 indicates good results, 0.7 to 0.8 indicates fair results, 0.6 to 0.7 indicates poor results, and 0.5 to 0.6 indicates failure of the results [32].

ArcGIS (10.8) software was used to visualize a predicted distribution map of the species [37]. Habitat suitability was classified into four categories—high, medium, low, and unsuitable—using the natural breaks (Jenks) method. These were visualized with distinct colors: red (high suitability), yellow (medium), green (low), and white (unsuitable) [3].

### 2.5. Center of Gravity Migration

Climate change may drive species to migrate in search of more suitable habitats. To accurately track this phenomenon, the centroid (representing the geographic center of a species’ distribution) serves as a key indicator of spatial distribution shifts, reflecting habitat migration patterns. The SDMtoolbox in ArcGIS 10.8 was used to track changes in the centroid latitude and longitude, as well as migration distances, across different time periods and climate scenarios [38,39]. Additionally, the overall migration trend of the Chinese forest frog in response to climate change was analyzed.

## 3. Results

### 3.1. Validation and Comparison of Models

Based on the 127 distribution points and 24 environmental variables for the Chinese forest frog, the MaxEnt model was used to simulate and predict the potential distribution of Chinese forest frog habitats. The optimal model was identified when RM = 1, FC = LQHTP, and delta AICc = 0, according to the AIC (Table 2).

**Figure 2:**
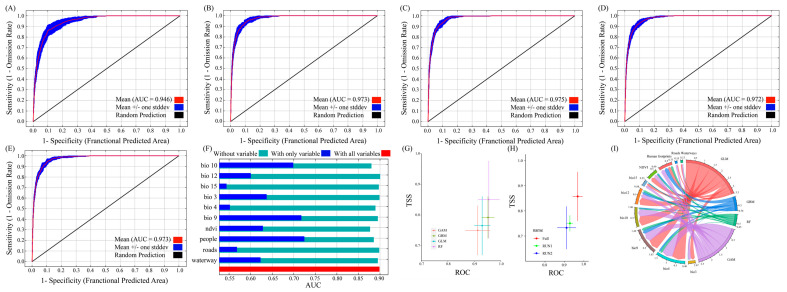
AUC values of the Biomod2 and MaxEnt models and jackknife test results for environmental variables. (**A**) ROC curve and AUC for the current period; (**B**) ROC curve and AUC for 2050s under SSP126; (**C**) ROC curve and AUC for 2050s under SSP585; (**D**) ROC curve and AUC for 2090s under SSP126; (**E**) ROC curve and AUC for 2090s under SSP585; (**F**) jackknife test results showing the relative contribution of environmental variables based on cross-validated AUC; (**G**,**H**) TSS and AUC values of the Biomod2 model across all time periods; (**I**) variable importance across different models, based on collinearity analysis.

MaxEnt and Biomod2 simulations for current and future (2050s and 2090s) scenarios were performed with optimal parameters, and the AUC results are shown in Figure 2. The values of the area under the receiver operating characteristic curve were all greater than 0.94. The TSS value shows that the model described the observed data correctly and exhibited good predictive performance (Figure 2G,H and Figure S3; Table 3). In summary, the optimized MaxEnt model and Biomod2 demonstrated high reliability in predicting the potential suitable habitats for the Chinese forest frog. The final model incorporated 10 environmental variables, including 6 climate factors, 2 distance-related factors, 1 vegetation index, and 1 anthropogenic factor (Table 3).

### 3.2. Importance of Environmental Variables

To evaluate the influence of environmental variables on habitat suitability, percent contribution (PC) and permutation importance (PI) were used as the key indicators in the MaxEnt model. In this study, the significance of the 10 predicted environmental variables was assessed using PC, PI, and jackknife tests. The MaxEnt results show that the temperature of the driest quarter (Bio9; 7.4%, 6.2%, and 71.8%), the temperature of the warmest quarter (Bio10; 28.1%, 18.6%, and 69.95%), and the human footprint (people; 19.4%, 18.6%, and 72.51%) were the most important environmental factors affecting the distribution of the Chinese forest frog (Figure 2 and Figure S2; Table 4). The Biomod2 results show that temperature seasonality, the mean temperature of the driest quarter, and the mean temperature of the warmest quarter were the most important environmental factors affecting the distribution of the Chinese forest frog (Figure 2I and Figure S3K).

**Figure 3:**
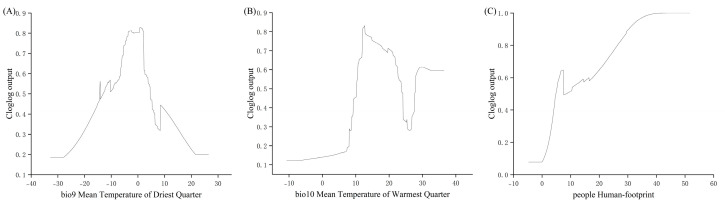
Response curves of selected environment variables generated by MaxEnt. (**A**) Bio9, mean temperature of driest quarter; (**B**) Bio10, mean temperature of warmest quarter; (**C**) People, human footprint.

Based on the response curves of the key environmental variables (Figure 3), the suitable environmental ranges for the Chinese forest frog were identified according to the predicted occurrence probabilities derived from the MaxEnt model. Specifically, suitable conditions were defined as those within the range of low-to-high-suitability values. The estimated suitable range for the temperature of the driest quarter was –37.77 to 26.46 °C; for the temperature of the warmest quarter, it was –10.69 to 36.48 °C; and for the human footprint index, it was 4.7 to 51.7.

### 3.3. Potential Distribution

The Biomod2 model revealed some differences in the distribution regions of the Chinese forest frog (Figure S1). However, the results show that the optimized MaxEnt model was the most consistent with the actual distribution. Therefore, the MaxEnt model adjusted using the ENMeval package was chosen for the final modeling.

**Figure 4:**
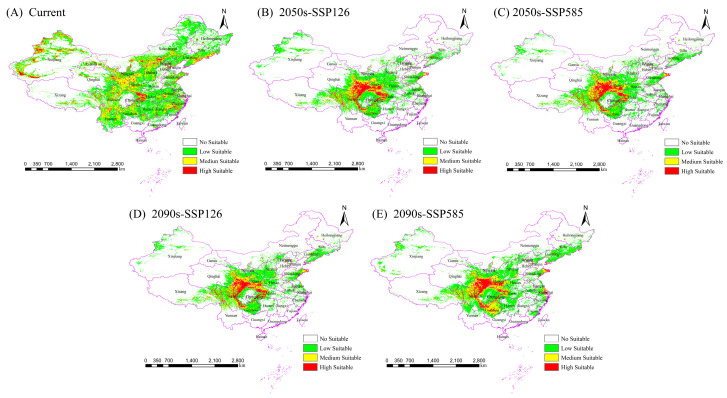
Predicted and confirmed distribution sites of current and future suitable habitats of the Chinese frog. (**A**) The current potential distribution. (**B**,**C**) The potential distributions under SSP126 and SSP585 in the 2050s. (**D**,**E**) The potential distributions under SSP126 and SSP585 in the 2090s.

The current potential suitable distribution areas for the Chinese forest frog are shown in Figure 4A. Under the current climatic conditions, the potential suitable habitat for the Chinese forest frog spans multiple provinces in China, including Heilongjiang, Jilin, Liaoning, Hebei, Shandong, Shanxi, Shanxi, Ningxia, Gansu, Xinjiang, Sichuan, Chongqing, Guizhou, Hunan, Hubei, Jiangxi, Zhejiang, Jiangsu, and Anhui. Currently, the total area of suitable habitat in China for the Chinese forest frog is 426 × 10^4^ km^2^, with medium- and high-suitability habitat areas covering 126 × 10^4^ km^2^ and 33 × 10^4^ km^2^, respectively, accounting for about 37.32% of the total distribution area (Table 5). The low-suitability habitat area is 267 × 10^4^ km^2^, accounting for about 62.68% of the total distribution area (Table 5). The high-suitability areas are mainly concentrated in Chongqing, Hubei, Xinjiang, and several other provinces in China.

The potential distribution of the Chinese forest frog under future climate scenarios (SSP126 and SSP585) in the 2050s and 2090s was projected and compared with the current distribution pattern (Figure 4B–E and Table 4). In the future, the distribution of the Chinese forest frog is predicted to be more concentrated, mainly in central and eastern China. Compared with the current scenario, the potential total distribution area and different levels of suitable areas for the Chinese forest frog are expected to be greatly reduced under SSP126. However, under the SSP585 scenario, the distribution area is predicted to first decrease and then increase, with the overall trend continuing to decrease.

### 3.4. Potential Changes in the Distribution Under Future Scenarios

**Figure 5:**
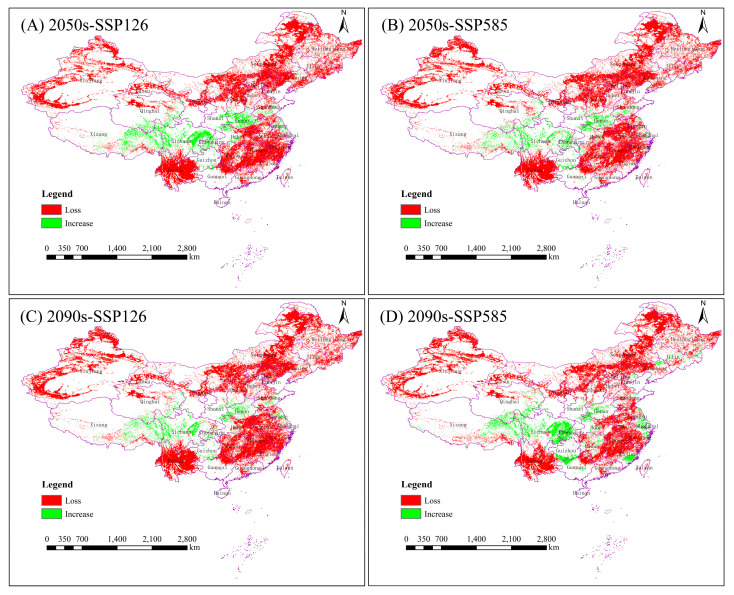
Spatial shifts in suitable habitats for the Chinese forest frog across different climate scenarios. (**A**) Lowest greenhouse gas emission scenario (SSP126) in 2041–2060; (**B**) highest-emission scenario (SSP585) in 2041–2060; (**C**) lowest-emission scenario (SSP126) in 2081–2100; and (**D**) highest-emission scenario (SSP585) in 2081–2100. Changes in suitable areas (gains and losses) are shown relative to the current distribution.

Comparative analyses of changes in the spatial pattern of potential suitable areas for the Chinese forest frog under four future climate scenarios were conducted based on the current potential distribution results (Figure 5 and Table 6). Under future climate scenarios, significant decreases in the suitable habitat for the Chinese forest frog are expected to occur. Under the four future climatic scenarios, the rate of increase in the suitable habitat for the Chinese forest frog ranges from 2.03 to 3.72%, concentrated in the provinces of Sichuan, Henan, Xizang and Chongqing, and the rate of loss ranges from 22.52 to 25.71%, concentrated in the provinces of Yunnan, Xinjiang, Jiangxi, Heilongjiang and Neimenggu. The percentage reduction in the suitable habitat area increased from 21.91% to 23.68% in the SSP126 scenario and decreased from 23.29% to 18.8% in the SSP585 scenario. The future 2090 high-emission scenario (SSP585) is expected to have the lowest reduction in the area of suitable habitat (Table 6).

**Figure 6:**
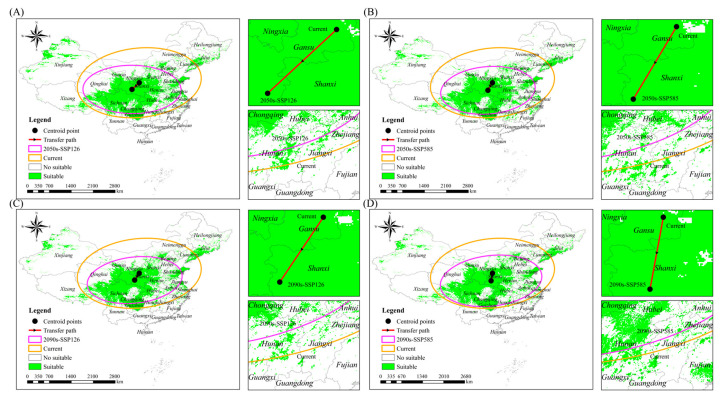
Centroid position of Chinese forest frog under different climate scenarios. (**A**) Current scenario and 2050s-SSP126; (**B**) current scenario and 2050s-SSP585; (**C**) current scenario and 2090s-SSP126; (**D**) and current scenario and 2090s-SSP585.

As the shape of the suitable range is irregular, the center of mass (centroid) was used to characterize the geography of the Chinese forest frog’s suitable range under different climate change scenarios. Under the current climatic conditions, the centroid is located in Gansu province (35°55′46″ N, 108°05′52″ E; Figure 6 and Table 7). In the SSP126 scenario, the centroid shifts southwestward in the 2050s (34°00′41″ N, 106°01′05″ E; Figure 6A and Table 7) and continues migrating southwestward in the 2090s (34°29′37″ N, 106°42′28″ E; Figure 6C and Table 7). Under the SSP585 scenario, the centroid similarly shifts southwestward into Shanxi province in the 2050s (33°45′02″ N, 106°47′38″ E; Figure 6B and Table 7) and moves further southwest in the 2090s (33°19′30″ N, 106°17′34″ E; Figure 6D and Table 7). These results indicate a consistent southwestward shift in the centroid of suitable habitats under both emission scenarios, with a more pronounced displacement observed under SSP585.

## 4. Discussion

### 4.1. Environmental Variable Predictors and Model Performance

SDMs can be categorized into three types: mechanistic, regression, and ecological niche models. Among these, ecological niche models, which utilize data from known distribution sites and relevant environmental factors, offer more reliable projections of species’ actual and potential distributions [40]. The MaxEnt model is a widely used method for ecological niche modeling. To enhance the model’s accuracy, feature combinations were optimally selected using the ENMeval package in R (version 4.1.3), reducing overfitting and sampling bias. In this study, five numerical regularization multipliers (ranging from one to five) and six feature combinations (L, LQ, H, LQH, LQHP, and LQHPT) were tested, resulting in thirty parameter combinations. The optimal configuration, with FC = LQHTP and RM = 1, yielded a delta AICc of 0 and an AUC of 0.946, demonstrating the model’s accuracy and reliability in simulating the potential distribution of the Chinese forest frog. Most existing studies on species distribution typically rely on climate factors or a combination of climate, terrain, and soil factors, often using default parameters or optimizing only the MaxEnt parameters in climate-based models [30]. Such approaches tend to overfit and increase model complexity, which can reduce accuracy and produce unreliable results, such as highly volatile environmental response curves. Therefore, model optimization is crucial for improving predictive performance.

The MaxEnt model identified the mean temperature of the driest quarter (Bio9), the mean temperature of the warmest quarter (Bio10), and the human footprint index as the most influential environmental predictors of the Chinese forest frog distribution. As ectotherms, amphibians exhibit strong habitat selection dependencies on thermal regimes and hydric conditions [41]. Although precipitation variables dominate Ranidae distribution models in published studies—for example, the range limitation of *Rana hanluica* by the precipitation of the driest month [42]—this discrepancy may reflect scale-dependent non-stationarity in environmental drivers, as hypothesized in macroecological frameworks [43]. In contrast, our multi-scale analysis establishes temperature as the consistent predictor across spatial grains. Notably, the temperature-driven distribution patterns observed here mirror those reported for *Hoplobatrachus chinensis* [44]. Among the environmental variables used in the model, temperature variation had the highest contribution (44.1%) to the species distribution.

The Chinese forest frog primarily occupies northern China’s warm temperate zone (Eurasian continental climate) with marked seasonality, a distribution pattern strongly supported by our model predictions [23]. Thermal constraints critically mediate amphibian physiological thresholds, directly impacting survival rates and reproductive outcomes [45]. Under warming scenarios, elevated temperatures disrupt developmental trajectories through dual mechanisms such as increased metabolic oxygen demand and decreased dissolved oxygen saturation in aquatic environments, creating physiological bottlenecks for larval stages [46,47,48]. Behavioral thermoregulation strategies, including altitudinal migration and microhabitat selection, represent key adaptive responses to thermal stress [49].

Species identification challenges arise during tadpole stages due to limited morphological diagnosability, while adults predominantly inhabit montane forest ecotones (grassy/shrubby understories), resulting in observational bias toward terrestrial habitats distant from aquatic systems. Breeding phenology further restricts detection opportunities, as adults only temporarily occupy aquatic breeding sites (e.g., ephemeral ponds and rice paddies). Interestingly, the MaxEnt model showed a positive correlation between human footprint index values and predicted species presence. This unexpected association may be influenced by model limitations or spatial data artifacts, such as indirect correlation with modified landscapes (e.g., irrigated farmlands), which provide potential but unconfirmed reproductive habitats. Further field-based validation is needed to assess the ecological plausibility of this pattern.

### 4.2. Response of the Spatial Distribution Pattern of the Chinese Forest Frog to Climate Change

Under future climate scenarios, the potential suitable habitat of the Chinese forest frog largely overlaps with its current distribution, which is primarily concentrated in the mid-temperate, southern temperate, mid-subtropical, northern subtropical, and plateau regions. An analysis of habitat shifts indicates a noticeable westward and southward migration of its primary suitable habitats. Similar to findings in other studies, this trend suggests that, under global warming, high-altitude and cool mountainous regions may provide more favorable habitats [47].

In this study, both high- and low-emission scenarios project a significant reduction in the potential suitable habitat of the Chinese forest frog, with an estimated habitat loss ranging from 22.52% to 25.71%. Notably, the lost areas predominantly correspond to regions classified as moderately or marginally suitable in the predictions. Consistent with trends observed in European amphibians [9,12], ongoing climate warming is highly likely to erode Chinese forest frog habitats, posing an increasingly severe threat to its survival.

Addressing climate change in the future presents a serious challenge. As global climate patterns continue to shift, the risk of substantial habitat contraction for the Chinese forest frog increases, presenting a significant challenge for the conservation of its wild populations. Given its widespread distribution across China, the decline in Chinese forest frog populations could have detrimental effects on amphibian diversity and overall ecological stability. Therefore, it is crucial to implement conservation strategies, such as habitat protection and restoration, the establishment of microclimate and microhabitat refugia, and water resource management to mitigate habitat loss and support the long-term survival of the Chinese forest frog.

## 5. Conclusions

In this study, occurrence data from 127 distribution points and 10 selected environmental variables were utilized to predict potential changes in the distribution of the Chinese forest frog under four future climate scenarios using an optimized MaxEnt model. Additionally, key environmental factors influencing its distribution were identified. The results indicate that under future climate change, the Chinese forest frog is projected to exhibit a westward and southward expansion trend, although its potential distribution is at risk of contraction. The primary environmental factors influencing its distribution include the mean temperature of the driest quarter, the mean temperature of the warmest quarter, and the human footprint.

## Figures and Tables

**Table 1:** List of all environmental factors.

Environmental Variables	Description	Unit
Bio1	Annual mean temperature	°C
Bio2	Mean diurnal range	°C
Bio3	Isothermality	-
Bio4	Temperature seasonality	-
Bio5	Max temperature of warmest month	°C
Bio6	Min temperature of coldest month	°C
Bio7	Temperature annual range	°C
Bio8	Mean temperature of wettest quarter	°C
Bio9	Mean temperature of driest quarter	°C
Bio10	Mean temperature of warmest quarter	°C
Bio11	Mean temperature of coldest quarter	°C
Bio12	Annual precipitation	mm
Bio13	Precipitation of wettest month	mm
Bio14	Precipitation of driest month	mm
Bio15	Precipitation seasonality	-
Bio16	Precipitation of wettest quarter	mm
Bio17	Precipitation of driest quarter	mm
Bio18	Precipitation of warmest quarter	mm
Bio19	Precipitation of coldest quarter	mm
Ele	Altitude	m
People	Human footprint	-
NDVI	Mean normalized vegetation index value, 2010–2019	-
Waterway	Euclidean distance to waterways	-
Roads	Euclidean distance to roads	-

**Table 2:** Evaluation metrics of MaxEnt model generated by ENMeval.

Type	FC	RM	Delta AICc	Avg. Diff. AUC
Default	LQHTP	1	0	0.07957
Optimized	LQHTP	1	0	0.07957

Note: FC: feature combination; RM: regulatory multiplier; LQTHP: linear features (L) + quadratic features (Q) + threshold (T) + hinge features (H) + product features (P); delta AICc: minimum information criterion AICc value; Avg. Diff. AUC: difference between AUC values.

**Table 3:** Evaluated TSS values of the model for each period.

Period	Current	2050s-SSP126	2050s-SSP585	2090s-SSP126	2090s-SSP585
Mean ± SD	0.641 ± 0.0567	0.816 ± 0.0353	0.835 ± 0.0314	0.825 ± 0.0251	0.842 ± 0.0168

**Table 4:** Summary of key environmental variables influencing the distribution of the Chinese forest frog.

Environmental Variables	Description	Unit	PC (%)	PI (%)
Bio3	Isothermality	-	10.1	9.8
Bio4	Temperature seasonality	-	5	8.5
Bio9	Mean temperature of driest quarter	°C	7.4	6.2
Bio10	Mean temperature of warmest quarter	°C	28.1	18.6
Bio12	Annual precipitation	mm	7.3	12.7
Bio15	Precipitation seasonality	-	7.8	4.2
People	Human footprint		19.4	18.6
NDVI	Mean normalized vegetation index value from 2010 to 2019	-	11.5	17.2
Waterway	Euclidean distance to waterways	-	3.4	4
Roads	Euclidean distance to roads	-	0.1	0.1

**Table 5:** Area statistics of suitable areas of Chinese forest frog under different climate scenarios (unit: ×10^4^ km^2^).

Periods	Low Suitability		Medium Suitability		High Suitability		All Suitability Areas	
	Area	Percentage	Area	Percentage	Area	Percentage	Area	Percentage
Current	267	62.68	126	29.58	33	7.75	426	44.34
2050s-SSP126	143	66.51	46	21.40	26	12.09	215	22.43
2050s-SSP585	131	64.53	45	22.17	27	13.30	203	21.15
2090s-SSP126	129	65.15	44	22.22	25	12.63	198	20.66
2090s-SSP585	160	65.31	58	23.67	27	11.02	245	25.54

Note: The area percentages represent the ratios of different levels of suitable areas to the total suitable area under each climate scenario. The proportion of all suitable areas was calculated relative to the total national area (960 × 10^4^ km^2^).

**Table 6:** Spatial variation in suitable habitat for Chinese forest frog in different periods.

Periods	Area (×10^4^ km^2^)			Rate of Change (%)		
	Gain	Loss	Change	Gain	Loss	Change
2050s-SSP126	31	241	−210	3.21	25.12	−21.91
2050s-SSP585	22	246	−224	2.29	25.58	−23.29
2090s-SSP126	19	247	−227	2.03	25.71	−23.68
2090s-SSP585	36	216	−180	3.72	22.52	−18.80

Note: The area percentages represent the ratios of different levels of suitable areas to the total suitable area under various climate scenarios. The proportion of all suitable areas was calculated relative to the total national area (960 × 10^4^ km^2^).

**Table 7:** Centroid coordinates and migration distances.

Period	Climate Scenario	Longitude	Latitude	Migration Distance (×10^4^ km)
Current	-	108°05′52″ E	35°55′46″ N	-
2050s	SSP126	106°01′05″ E	34°00′41″ N	2.83
	SSP585	106°47′38″ E	33°45′02″ N	2.54
2090s	SSP126	106°42′28″ E	34°29′37″ N	2.00
	SSP585	106°17′34″ E	33°19′30″ N	3.17

## Data Availability

The original contributions presented in this study are included in the article/Supplementary Materials. Further inquiries can be directed to the corresponding authors.

## Supplementary Materials

The following supporting information can be downloaded at https://www.mdpi.com/article/10.3390/biology14070754/s1: Figure S1: Predicted and confirmed distribution sites of contemporary and future suitable habitats of the Chinese forest frog by Biomod2; Figure S2: Jackknife diagrams for different future scenarios; Figure S3: Plot of ROC and TSS values and environmental weights under different time periods. Current (A,B); 2050s-SSP126 (C,D); 2050s-SSP585 (E, F); 2090s-SSP126 (G,H); 2090s-SSP585 (I,J); and environment importance (K).
