# Supplementary material for: Predictions of the Chinese Forest Frog (Rana chensinensis) Distribution Pattern Under Climate Change up to 2090s
Source: Biology (Basel). 2025 Jun 24;14(7):754. doi: 10.3390/biology14070754 (PMC12292927; doi:10.3390/biology14070754)

Figure S1 *Predicted and confirmed distribution sites of contemporary and future suitable habitats of Chinese forest frog by Biomod2*

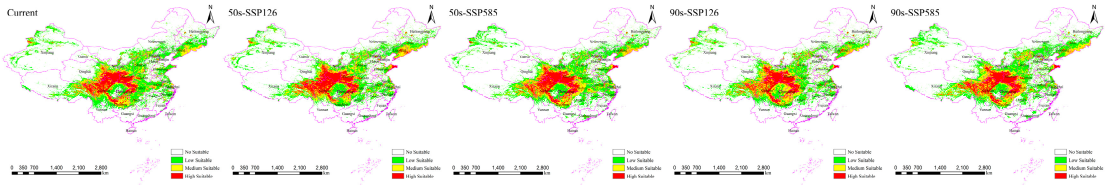

Figure S2 *Jackknife diagrams for different future scenarios*

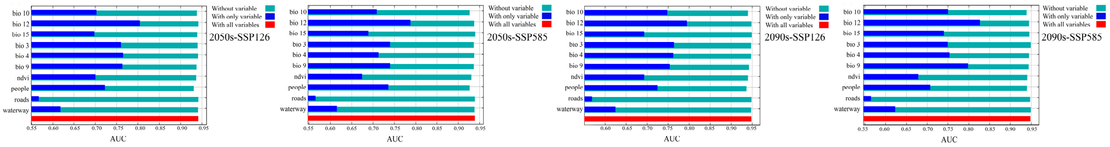

Figure S3 *Plot of ROC and TSS values and environmental weights under different time periods. Current (A, B); 2050s-SSP126 (C, D); 2050s-SSP585 (E, F);2090s-SSP126 (G, H); 2090s-SSP585 (I, J); Environment important (K).*

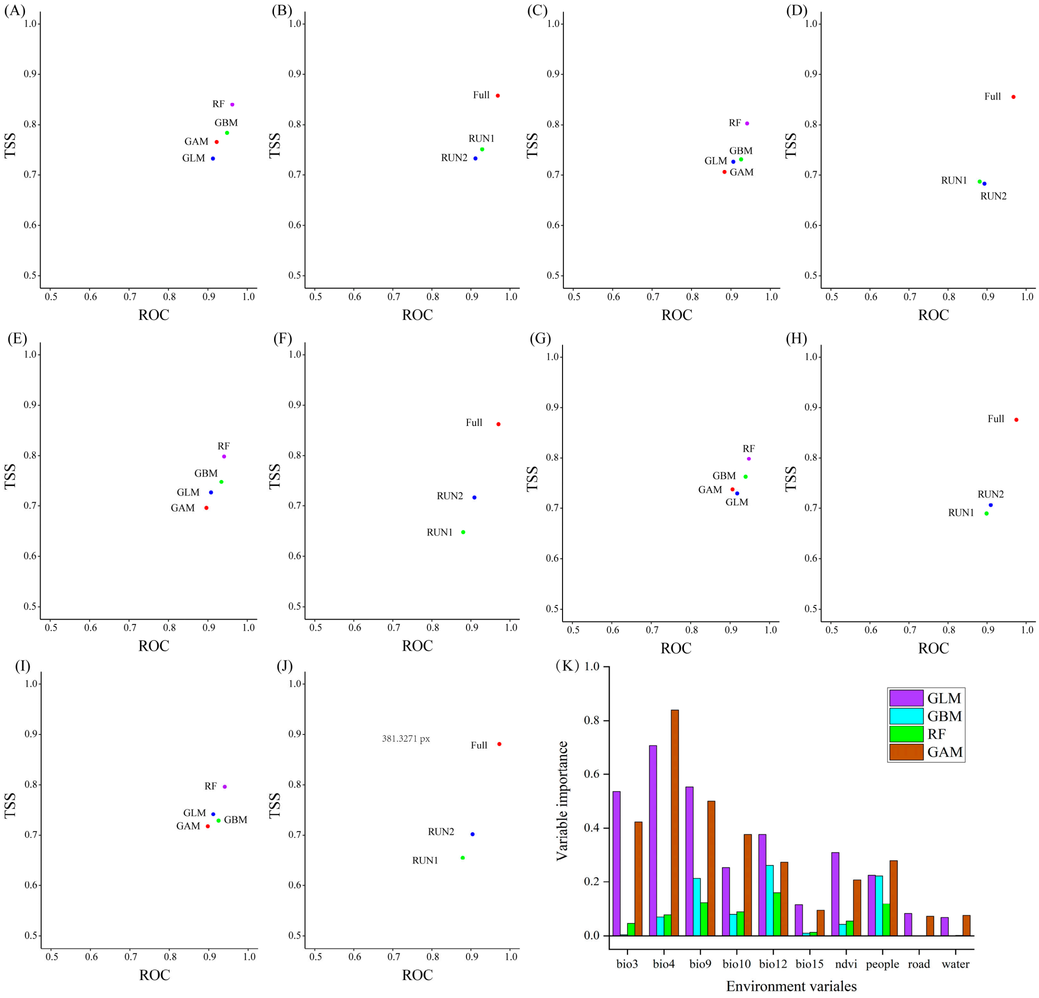

Supplement: Supplementary file 1 [file biology-14-00754-s001.zip › figure-S.pdf]
